# Bespoke extensional elasticity through helical lattice systems

**DOI:** 10.1098/rspa.2019.0547

**Published:** 2019-12-04

**Authors:** Maximillian D. X. Dixon, Matthew P. O'Donnell, Alberto Pirrera, Isaac V. Chenchiah

**Affiliations:** 1 Bristol Composites Institute (ACCIS), Department of Aerospace Engineering, University of Bristol, Bristol BS8 1TR, UK; 2 School of Mathematics, University of Bristol, Bristol BS8 1UG, UK

**Keywords:** nonlinear spring, bespoke stiffness, lattice, metamaterials, anisotropy, multi-stability

## Abstract

Nonlinear structural behaviour offers a richness of response that cannot be replicated within a traditional linear design paradigm. However, designing robust and reliable nonlinearity remains a challenge, in part, due to the difficulty in describing the behaviour of nonlinear systems in an intuitive manner. Here, we present an approach that overcomes this difficulty by constructing an effectively one-dimensional system that can be tuned to produce bespoke nonlinear responses in a systematic and understandable manner. Specifically, given a continuous energy function E and a tolerance *ϵ* > 0, we construct a system whose energy is approximately E up to an additive constant, with L^∞^-error no more that *ϵ*. The system is composed of helical lattices that act as one-dimensional nonlinear springs in parallel. We demonstrate that the energy of the system can approximate any polynomial and, thus, by Weierstrass approximation theorem, any continuous function. We implement an algorithm to tune the geometry, stiffness and pre-strain of each lattice to obtain the desired system behaviour systematically. Examples are provided to show the richness of the design space and highlight how the system can exhibit increasingly complex behaviours including tailored deformation-dependent stiffness, snap-through buckling and multi-stability.

## 1. Introduction

Historically, elastic nonlinearities have been avoided by structural engineers. This is often due to the need to simplify the design process and limit the sensitivity to design parameters in order to create robust, repeatable behaviour. Moreover, elastic nonlinearities are often synonymous with sudden catastrophic failure. However, recent advances in material construction processes, e.g. three-dimensional printing, and the availability of advanced computational/experimental analysis methods have permitted a relaxation of this design conservatism [1–5]. In particular, computation has permitted the probing of complex nonlinear design spaces with resultant improvements such as reduced structural weight, increased robustness or additional functionality [2,6].

In general, the potential for multifunctional designs to reduce system part count is particularly attractive from the perspectives of structural weight and minimal design philosophy. However, a trade-off ensues. Conventional components are stiff and movement is achieved through mechanical joints, such as hinges. To eliminate mechanical joints but retain operational load-bearing capacity, greater control of stiffness and form is required at both global and local structural levels [7]. In this context, nonlinear elastic deformations are an attractive means for robust and repeatable shape and stiffness adaptation. The desire to explore and exploit nonlinearity has motivated more complex descriptions of the underlying mechanics. For instance, with structures capable of large deformations, geometrically nonlinear stress analysis is often involved in the design process [8–11].

An emerging and somewhat more challenging task than describing the nonlinear response of a structure under loading is its inverse, i.e. designing a structural topology and material system to exhibit a specific nonlinear response. The need for bespoke elastic responses has arisen in many fields including energy absorption [12], robotic and MEMS actuation [8,13,14], extreme thermoelastic devices [15] and flow regulation valves [6,16]. The strength of the nonlinearity in these bespoke responses is often limited so it can be realized and manufactured. In cases where the nonlinearity is unconstrained, a discussion of how such responses can be realized with current design and manufacturing technologies is often omitted. In the instances where physical systems have been fabricated, they are of little use outside of the intended application [12]. Therefore, a universal design framework able to achieve an arbitrary nonlinear response in a systematic manner would be immensely valuable to the wider engineering community.

Bespoke nonlinear elasticity has already been investigated by some authors. Typically, the focus has been on finding and coupling suitable problem parametrizations with nonlinear finite-element analysis (FEA) solvers and optimization algorithms [8–11]. Due to the complexity of the underlying structural models, the process of tailoring nonlinear responses has often been reported as computationally expensive. As an example, Jutte [17] successfully used topology optimization, coupled with a nonlinear FEA solver, on a two-dimensional network of geometrically nonlinear curved beams. The level of achievable nonlinearities was restricted though, with buckling or snap-through behaviours excluded. A variety of nonlinearities were observed, including constant and nonlinear strain hardening and softening curves.

**Figure 1.**
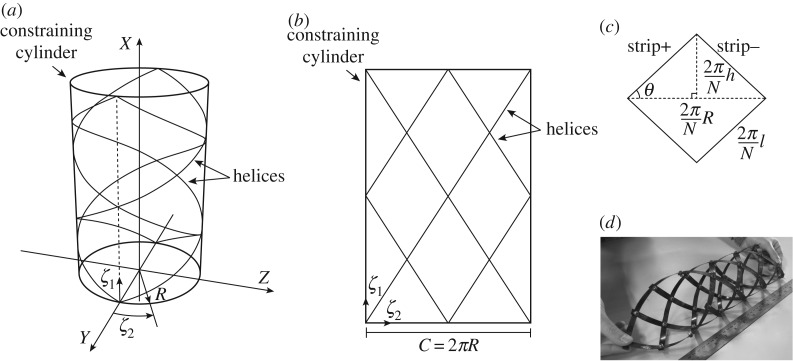
(*a*) A cylindrical lattice, (*b*) planar projection of lattice, (*c*) representative unit cell and (*d*) composite prototype. Sketch of a typical lattice comprising four helical strips (i.e. N = 2) on the surface of its constraining cylinder and its planar representation. Adapted from [18].

In the context of nonlinear lattice structures, Pirrera *et al.* [18] inspired by the behaviour of the virus bacteriophage T4, explored the design space of geometrically frustrated helical lattices (figure 1*d*) demonstrating robust, elastic multi-stability. Similar structures, with a focus on the handedness of their auxetic properties, were investigated by Lipton *et al.* [19]. Also of note is the work on gridshells by Baek *et al.* [20,21], where the authors investigate the shape and rigidity of shell-like structures arising from the buckling of initially planar grids of rods.

### (a) Main contributions of this article

To the authors' knowledge, there are currently no tools capable of designing a one-dimensional system to exhibit arbitrary nonlinear extensional responses. We address this deficiency by presenting a system that can be constructed as an assembly of well-understood nonlinear, spring-like ‘units’. The units' behaviour is described by means of a simple analytical model, which provides insight into the system's mechanics while requiring minimal computational effort for design. As units are added to the assembly, new aspects of behaviour may be observed. In essence, as we increase the complexity of the system, richer responses can be achieved without increasing the complexity of the description of the system. Following this mechanism, we are able to design nonlinear spring-like systems that can achieve a plethora of desirable response characteristics.

In the present work, the fundamental units are helical lattices like that illustrated in figure 1. This structure was first presented in [18], where robust nonlinear features, such as multi-stability, were observed for various ranges of lattice geometry, stiffness and pre-strain. This lattice acts as an effective one-dimensional spring, the behaviour of which may be described succinctly using its energetic profile under extension. Here, multiple helical lattices are coupled through rigid connections, in arrangements similar to those presented in [19] whilst preserving the kinematic assumption outlined in [18]. We demonstrate the appearance of new response characteristics that are unobtainable via a single lattice in isolation. Besides, further behaviour continues to emerge with additional geometrically distinct lattices.

Mathematically, linking lattices changes the system's energy in a manner that resembles a series expansion. We demonstrate that this offers sufficient design freedom to approximate any polynomial energy, and thus to approximate any continuous energy to any desired accuracy in L^∞^. In particular, we can prescribe the positions and stability of the system's equilibria, as well as its stiffness as a function of extension. In short, we can design a bespoke nonlinear elastic response. We present a systematic approach to obtaining the design parameters for individual lattices and the overall system assembly requirements.

### (b) Outline

The remainder of paper proceeds as follows. In §§2 and 3, we first recall and then recast the results of Pirrera *et al.* [18] for a single lattice. In §4, we extended these results to coupled systems of multiple lattices. In §5, we prepare the ground for the main result which is then proved in §6. Ancillary results are presented in §7. Several examples of engineered behaviours illustrating the robustness of the design space and the algorithm are presented in §8. Finally, conclusion is drawn in §9.

## 2. Kinematics and elastic energy of single lattices

Let us consider a generic helical lattice—as depicted in figure 1*a*—consisting of N pairs of anisotropic helices, kinematically constrained to lie upon the surface of a reference cylinder of radius R. Pirrera *et al.* [18] developed an analytical formulation to describe the mechanics of such a system, based on the elastic energy of the helical strips. For the reader's convenience, we repeat the kinematic analysis [15] here.

The lattice can extend or compress as a result of loading at its extremities and along the longitudinal axis (i.e. the X-direction). As the lattice deforms, we assume [15,18] that:

(i) all helices remain on a single cylinder (whose radius and length can change);
(ii) the helical strips are hinged where they overlap allowing scissoring motion only;
(iii) the change in length of the helical strips is negligible; and
(iv) the helical strips are sufficiently slender that the dominant contribution to the energy arises from bending and twisting with respect to the strips' principal dimension.

Furthermore, consider the ‘+’ strip that passes through the origin in figure 1*b*. In the *ζ*-plane, this strip has the parametrization

$$(\zeta_{1},\zeta_{2})=s(\sin(\theta ),\cos(\theta )),$$

where s is the arc-length parameter and *θ* is illustrated in figure 1*c*. From the assumptions above, this strip maps to the helix

2.1 $$\gamma =\zeta_{1}\hat{X}+RQ\left(\frac{\zeta_{2}}{R}\right)\hat{Y}=s\sin(\theta )\hat{X}+RQ\left(\frac{\cos(\theta )}{R}s\right)\hat{Y},$$

in the (X, Y, Z) coordinate system used in figure 1*a*, where a hat is used to indicate unit vectors along these axes. **Q**(*ϕ*)∈SO(3) is a rotation about $\hat{X}$ through an angle *ϕ*. The unit tangent **t**(s), normal **n**(s) and binormal **b**(s) are defined as

$$\begin{array}{rl} & t(s)=\frac{\gamma^{\prime }}{∥\gamma ∥}=\sin(\theta )\hat{X}+\cos(\theta )Q\left(\frac{\cos(\theta )}{R}s\right)\hat{Z},\\ & n(s)=\frac{t^{\prime }}{∥t^{\prime }∥}=-Q\left(\frac{\cos(\theta )}{R}s\right)\hat{Y}\\ \;\mathrm{and}\; & b(s)=t(s)\times n(s)=\cos(\theta )\hat{X}-\sin(\theta )Q\left(\frac{\cos(\theta )}{R}s\right)\hat{Z}, \end{array}$$

where dash indicates differentiation with respect to the arc-length parameter, s. The unit cell extension,

2.2 h∈[0,l],

as shown in figure 1*c*, is used to uniquely describe the state of the system. Accordingly, the curvature *κ*_x_, and torsion *κ*_xy_, can be defined in terms of the unit cell extension, such that

2.3*a* $$\begin{array}{rl} \kappa_{x} & =t^{\prime }(s)\cdot n(s)=\frac{\cos^{2}(\theta )}{R}=\frac{\sqrt{l^{2}-h^{2}}}{2l^{2}} \end{array}$$

2.3*b* $$\begin{array}{rl} \;\text{and}\;\kappa_{xy} & =-b^{\prime }(s)\cdot n(s)=\frac{\sin(\theta )\cos(\theta )}{R}=\frac{h}{2l^{2}}. \end{array}$$

These definitions hold for the ‘−’ strip as well, except that the sign of the torsion is reversed. Since we have assumed that the strips' behaviour is dominated by bending and twisting deformations, i.e. the stretching energy can be neglected, as in [15,18], we obtain a quadratic bending energy of the form

2.4 $$U=\frac{1}{2}\left(\kappa^{⊤}d\kappa \right),$$

where the curvature components ***κ*** and associated reduced bending stiffness matrix **d** are described by Classical Laminate Theory (CLT) [22] capturing stiffness anisotropy. The use of CLT is for notational convenience alone and does not imply that the system *must* be composed of composite strips. For example, anisotropy could be designed into the system by exploiting cross-sectional geometry or functionally graded materials.

## 3. Unit cell formulation

Figure 1 illustrates the lattice geometry schematically, its development into a plane, and the representative unit cell upon which the lattice behaviour can be modelled. Herein, we limit right- and left-handed helices—denoted by subscripts · _+_ and · _−_, respectively—to have equal and opposite pitch, *θ*. This restriction ensures unit cells are rhombi and prevents the lattice from twisting upon extension [18]. In addition, and conveniently for the current purposes, it creates a bijective correspondence between R and h [18], permitting the lattice extension to be used as the single degree of freedom to characterize the system [23].

### (a) Total potential energy of a unit cell

With the unit cell representation, it is sufficient to consider the total potential energy of the constitutive cell to describe overall lattice behaviour. For simplicity, but without loss of generality, it is assumed that each right-handed helix has identical dimensions, stiffness and mechanical pre-strain and that these properties are constant along the length of the helices. A similar commonality is shared between left-handed helices. Using (2.4), the energy of the representative unit cell can then be written as

3.1 $$\Pi =\frac{\pi l}{N}\left(w_{+}{\left(\kappa_{+}-\upsilon_{+}\right)}^{⊤}d_{+}(\kappa_{+}-\upsilon_{+})+w_{-}{\left(\kappa_{-}-\upsilon_{-}\right)}^{⊤}d_{-}(\kappa_{-}-\upsilon_{-})\right),$$

where (2*π*l/N) is the cell's side length and w_±_ the strip's width. The model by Pirrera *et al.* [18] considers axial, · _x_, and twist, · _xy_, components of curvature. Moreover, the total curvature comprises two additive terms: the curvature developed by the helices upon deformation while conforming to the surface of the cylinder, ***κ***_±_(h), and the mechanical pre-curvature (tooling), ***υ***_±_. Hence,

3.2 $$\kappa_{\pm }(h)-\upsilon_{\pm }=\left[\begin{array}{c} \kappa_{x\pm }(h)\\ \kappa_{xy\pm }(h) \end{array}\right]-\left[\begin{array}{c} \upsilon_{x\pm }\\ \upsilon_{xy\pm } \end{array}\right],$$

which is included in the energetic formulation with the corresponding terms of the reduced bending stiffness matrix, **d**_±_

3.3 $$d_{\pm }=\left[\begin{array}{cc} d_{11\pm } & d_{16\pm }\\ d_{16\pm } & d_{66\pm } \end{array}\right].$$

The curvatures due to lattice extension may be stated using the Weingarten map [24]

3.4 $$\kappa_{\pm }(h)=\frac{1}{R}\left[\begin{array}{c} \cos^{2}\theta \\ \pm \sin\theta \cos\theta \end{array}\right]=\frac{1}{2l^{2}}\left[\begin{array}{c} \sqrt{l^{2}-h^{2}}\\ \pm h \end{array}\right],$$

where, from figure 1

3.5 $$R=2\sqrt{l^{2}-h^{2}}.$$

Equation (3.1) now may be expressed in terms of non-dimensional parameters.

### (b) Non-dimensional form

To aid analysis, we make the following normalizations, indicated by an overbar:

3.6*a* $$\begin{array}{rl} \bar{h} & =\frac{h}{l}, \end{array}$$

3.6*b* $$\begin{array}{rl} {\bar{d}}_{\pm } & =\frac{d}{d_{11\pm }}=\left[\begin{array}{cc} 1 & {\bar{\epsilon }}_{\pm }\\ {\bar{\epsilon }}_{\pm } & {\bar{\delta }}_{\pm } \end{array}\right], \end{array}$$

3.6*c* $$\begin{array}{rl} {\bar{\kappa }}_{\pm } & =2l\kappa_{\pm }, \end{array}$$

3.6*d* $$\begin{array}{rl} {\bar{\upsilon }}_{\pm } & =2l\upsilon_{\pm } \end{array}$$

3.6*e* $$\begin{array}{rl} \;\mathrm{and}\;\bar{\varphi } & =\frac{w_{-}d_{11-}}{w_{+}d_{11+}}. \end{array}$$

This allows the energy of the representative unit cell, (3.1), to be recast as

3.7 $$\begin{array}{rl} \Pi (\bar{h}) & =\frac{\pi }{4}\frac{w_{+}d_{11+}}{Nl}\left(a_{0}+a_{1}\bar{h}+a_{2}{\bar{h}}^{2}+b_{1}\sqrt{1-{\bar{h}}^{2}}+b_{2}\bar{h}\sqrt{1-{\bar{h}}^{2}}\right)\\ & =\frac{\pi }{4}\frac{w_{+}d_{11+}}{Nl}\bar{\Pi }(\bar{h}), \end{array}$$

for h∈[0, 1], with

3.8*a* $$\begin{array}{rl} & a_{0}=\left(1+{\bar{\upsilon }}_{x+}^{2}+2{\bar{\epsilon }}_{+}{\bar{\upsilon }}_{x+}{\bar{\upsilon }}_{xy+}+{\bar{\delta }}_{+}{\bar{\upsilon }}_{xy+}^{2}\right)+\bar{\varphi }\left(1+{\bar{\upsilon }}_{x-}^{2}+2{\bar{\epsilon }}_{-}{\bar{\upsilon }}_{x-}{\bar{\upsilon }}_{xy-}+{\bar{\delta }}_{-}{\bar{\upsilon }}_{xy-}^{2}\right), \end{array}$$

3.8*b* $$\begin{array}{rl} & a_{1}=-2\left({\bar{\epsilon }}_{+}{\bar{\upsilon }}_{x+}+{\bar{\delta }}_{+}{\bar{\upsilon }}_{xy+}\right)+2\bar{\varphi }\left({\bar{\epsilon }}_{-}{\bar{\upsilon }}_{x-}+{\bar{\delta }}_{-}{\bar{\upsilon }}_{xy-}\right), \end{array}$$

3.8*c* $$\begin{array}{rl} & a_{2}=\left({\bar{\delta }}_{+}-1\right)+\bar{\varphi }\left({\bar{\delta }}_{-}-1\right), \end{array}$$

3.8*d* $$\begin{array}{rl} & b_{1}=-2\left({\bar{\upsilon }}_{x+}+{\bar{\epsilon }}_{+}{\bar{\upsilon }}_{xy+}\right)-2\bar{\varphi }\left({\bar{\upsilon }}_{x-}+{\bar{\epsilon }}_{-}{\bar{\upsilon }}_{xy-}\right) \end{array}$$

3.8*e* $$\begin{array}{rl} \;\mathrm{and}\; & b_{2}=2{\bar{\epsilon }}_{+}-2\bar{\varphi }{\bar{\epsilon }}_{-}. \end{array}$$

For feasible systems, the stiffness coefficients must guarantee the positive definiteness of ${\bar{d}}_{\pm }$ and thus we require

3.9*a* $$\begin{array}{rl} \bar{\varphi } & >0, \end{array}$$

3.9*b* $$\begin{array}{rl} {\bar{\delta }}_{\pm } & >0 \end{array}$$

3.9*c* $$\begin{array}{rl} \mathrm{and}\;{\bar{\epsilon }}_{\pm }^{2} & <{\bar{\delta }}_{\pm }. \end{array}$$

Although the definition of the strain energy used herein only considers curvatures due to mechanical loading and pre-strain, the description can be readily modified to include additional fields, such as thermal, electromagnetic, piezoelectric, pH and moisture [15]. The inclusion of such effects is straightforward, but beyond the scope of the current analysis. Inclusion of these effects can improve prediction of manufactured performance of similar structural architectures [25,26] and field-dependent curvature changes offer additional avenues to manipulate the energy landscape of the system. In other words, the fields can be used, either in isolation or synergistically, to enhance the tailorability of the lattice. For instance, both the equilibria and their characteristics of stability can be tuned to change with temperature, creating nonlinear cyclic thermal actuators or highly tuned coefficients of thermal expansion beyond what is achievable by traditional materials [15].

## 4. Coupled lattice system

Although, as demonstrated in [18], a single helical lattice may be tuned to exhibit many desirable nonlinear behaviours, it does not provide the designer with complete freedom to develop a complete set of smooth energy profiles as a function of lattice extension. To overcome this limitation, it is advantageous to consider the behaviour of multiple lattices coupled together. Through the coupling of unique lattices, new responses are obtainable that cannot be achieved by a single lattice in isolation. Developing this hierarchical system shows how, depending on length scale, the tuning of nonlinear substructures can be considered analogous to the development of architectured or meta-materials (see, for instance [27]).

### (a) Lattices in parallel with common extension

Henceforth, coupled lattices are constrained to have the same extension. For clarity, we present diagrams showing a two-lattice system, but analyses and results are for systems with I∈N lattices. (Here N denotes the positive integers.)

#### (i) Extension

**Figure 2.**
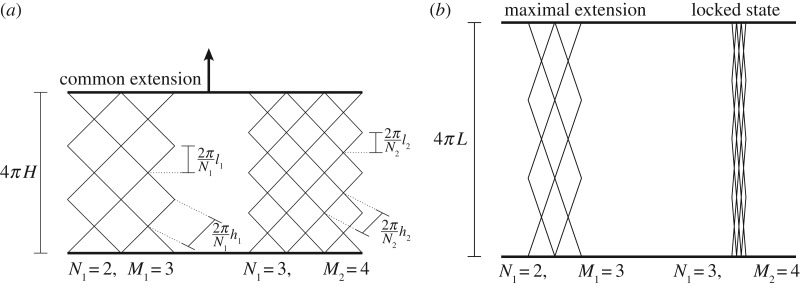
Plan form diagrams of a coupled lattice system comprising two lattices (cf. figure 1*b*). Showing (*a*) a partially extended state and (*b*) locking due to maximal extension of second lattice.

The ith lattice is composed of M_i_ bands of unit cells in the axial direction and N_i_ bands in the circumferential direction, as illustrated in figure 2. In this section, i = 1, 2, …, I, unless specified otherwise. From the geometry of the unit cell (figure 1), one can deduce that the maximum lattice extension is 4*π*L_i_, where

4.1*a* $$L_{i}=\frac{M_{i}l_{i}}{N_{i}}.$$

Since the lattice system locks when any lattice reaches full extension, as shown in figure 2*b*, the maximum possible extension of the system is 4*π*L, where

4.1*b* $$L=\min_{i}L_{i}.$$

Let the global extension of the system be 4*π*H, for H∈[0, L]. This global extension can be related to the extensions of the unit cells of individual lattices through

4.2*a* $$h_{i}=\frac{N_{i}H}{M_{i}}.$$

Then, the normalized extension of the ith lattice, ${\bar{h}}_{i}$ (see (3.6*a*)), can be related to the normalized extension of the system, $\bar{H}$, through a parameter *ψ*_i_:

4.2*b* $${\bar{h}}_{i}=\psi_{i}\bar{H},$$

where, using (4.1),

4.2*c* $$\psi_{i}=\frac{L}{L_{i}}=\frac{L}{l_{i}}\frac{N_{i}}{M_{i}}\in (0,1]$$

4.2*d* $$\;\text{and}\;\bar{H}=\frac{H}{L}\in [0,1].$$

#### (ii) Lattice energy

The energy of the ith lattice, *ξ*_i_, may be obtained by multiplying the energy of each unit cell, given by (3.7), and the number of unit cells, 2N_i_M_i_ (cf. figure 2*a*):

$$\xi_{i}(\bar{H})=\frac{\pi }{2}\frac{M_{i}}{l_{i}}w_{{+}_{i}}d_{11{+}_{i}}{\bar{\Pi }}_{i}(\psi_{i}\bar{H}),$$

where we have used (4.2*b*). This can be written as

4.3*a* $$\begin{array}{rl} \xi_{i}(\bar{H}) & =\Upsilon_{i}{\bar{\Pi }}_{i}(\psi_{i}\bar{H}) \end{array}$$

4.3*b* $$\begin{array}{rl} & =\Upsilon_{i}\left(a_{0i}+a_{1i}\psi_{i}\bar{H}+a_{2i}\psi_{i}^{2}{\bar{H}}^{2}+b_{1i}\sqrt{1-\psi_{i}^{2}{\bar{H}}^{2}}+b_{2i}\psi_{i}\bar{H}\sqrt{1-\psi_{i}^{2}{\bar{H}}^{2}}\right), \end{array}$$

4.4*a* $$\;\text{where}\;\Upsilon_{i}=\frac{\pi }{2}\frac{M_{i}}{l_{i}}w_{{+}_{i}}d_{11{+}_{i}},$$

and the coefficients a_0i_, a_1i_, a_2i_, b_1i_ and b_2i_ are related to the material parameters ${\bar{\delta }}_{i\pm }$, ${\bar{\epsilon }}_{i\pm }$, ${\bar{\upsilon }}_{ix\pm }$, ${\bar{\upsilon }}_{ixy\pm }$ and ${\bar{\varphi }}_{i}$ through (3.8). Note that

4.4*b* $$\Upsilon_{i}>0.$$

#### (iii) System energy

From (4.3), the system energy, *Ξ*, can be written as

4.5 $$\begin{array}{rl} \Xi (\bar{H}) & =\sum_{i=1}^{I}\xi_{i}(\bar{H})=\sum_{i=1}^{I}\Upsilon_{i}{\bar{\Pi }}_{i}(\psi_{i}\bar{H})\\ & =A_{0}+A_{1}\bar{H}+A_{2}{\bar{H}}^{2}+\sum_{i=1}^{I}B_{i}\;f_{\psi_{i}}(\bar{H})+\sum_{i=1}^{I}C_{i}\;g_{\psi_{i}}(\bar{H}), \end{array}$$

with coefficients

4.6*a* $$\begin{array}{rl} & A_{0}=\sum_{i=1}^{I}\Upsilon_{i}a_{0i}, \end{array}$$

4.6*b* $$\begin{array}{rl} & A_{1}=\sum_{i=1}^{I}\Upsilon_{i}a_{1i}\psi_{i}, \end{array}$$

4.6*c* $$\begin{array}{rl} & A_{2}=\sum_{i=1}^{I}\Upsilon_{i}a_{2i}\psi_{i}^{2}, \end{array}$$

4.6*d* $$\begin{array}{rl} & B_{i}=\Upsilon_{i}b_{1i} \end{array}$$

4.6*e* $$\begin{array}{rl} \;\mathrm{and}\; & C_{i}=\Upsilon_{i}b_{2i}, \end{array}$$

and functions f_*ψ*_, $g_{\psi }:[0,1]\to R_{+}$ given by

4.7*a* $$\begin{array}{r} f_{\psi }(\bar{H})=\sqrt{1-{\left(\psi \bar{H}\right)}^{2}} \end{array}$$

4.7*b* $$\begin{array}{rl} \;\text{and}\;g_{\psi }(\bar{H}) & =\psi \bar{H}f_{\psi }(\bar{H})=\psi \bar{H}\sqrt{1-{\left(\psi \bar{H}\right)}^{2}}. \end{array}$$

The A coefficients add no new behaviour to the lattice system compared to a single lattice since the summation merely replaces one constant with another. Functions of the form $B_{i}\;f_{\psi_{i}}(\bar{H})$ and $C_{i}\;g_{\psi_{i}}(\bar{H})$, however, contribute to the system energy with distinctive terms that a single lattice cannot possess. As we shall see, a generic lattice system will feature behaviour that cannot be reproduced by an individual lattice.

#### (iv) Stiffness and equilibria

Differentiating (4.7) with respect to $\bar{H}$, we obtain

$$\begin{array}{rl} f_{\psi }^{\prime }(\bar{H}) & =-\frac{\psi^{2}\bar{H}}{f_{\psi }(\bar{H})}=-\frac{\psi }{f_{\psi }(\bar{H})}\psi \bar{H}\\ \;\text{and}\;g_{\psi }^{\prime }(\bar{H}) & =-\frac{\psi^{3}{\bar{H}}^{2}}{f_{\psi }(\bar{H})}+\psi f_{\psi }(\bar{H})=\frac{\psi }{f_{\psi }(\bar{H})}\left(1-2\psi^{2}{\bar{H}}^{2}\right). \end{array}$$

Then, from (4.5), the first two derivatives of the normalized energy, $\bar{\Xi }$, with respect to extension are

4.8*a* $$\begin{array}{rl} \frac{d\Xi }{d\bar{H}} & =A_{1}+2A_{2}\bar{H}+\sum_{i}\frac{\Upsilon_{i}}{\sqrt{1-{\left(\psi_{i}\bar{H}\right)}^{2}}}\left(b_{2i}\psi_{i}-b_{1i}\psi_{i}^{2}\bar{H}-2b_{2i}\psi_{i}^{3}{\bar{H}}^{2}\right) \end{array}$$

4.8*b* $$\begin{array}{rl} \;\text{and}\;\frac{d^{2}\Xi }{d{\bar{H}}^{2}} & =2A_{2}+\sum_{i}\frac{\Upsilon_{i}}{{\left(1-{\left(\psi_{i}\bar{H}\right)}^{2}\right)}^{3/2}}\left(-b_{1i}\psi_{i}^{2}-3b_{2i}\psi_{i}^{3}\bar{H}+2b_{2i}\psi_{i}^{5}{\bar{H}}^{3}\right). \end{array}$$

The stiffness, equilibria and characteristics of stability of the system can be deduced from these expressions.

### (b) Concentric lattices

**Figure 3.**
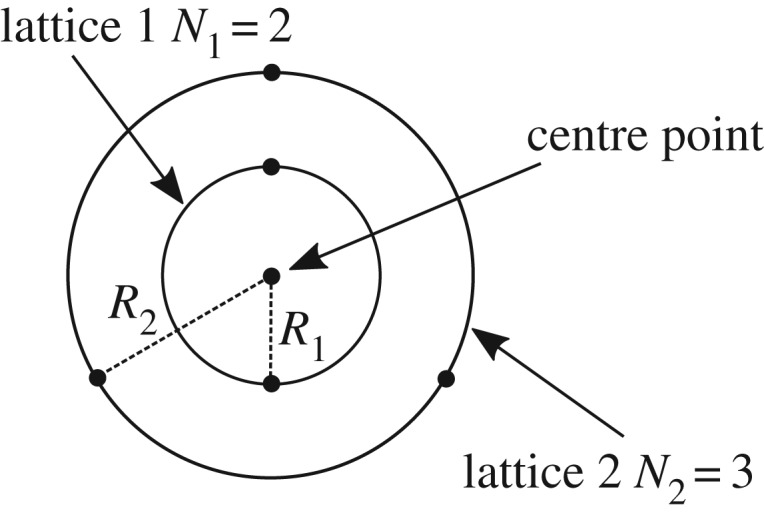
Plan view of concentric lattice system.

We can restrict the behaviour of the system by arranging the lattices in a concentric manner, as illustrated in figure 3. As the lattices are now concentric they are not permitted to intersect one another. In other words, when the lattices are numbered outwards from the centre, R_i_ ≤ slantR_i+1_.

Since, from (3.5), (4.1) and (4.2), the radius of the ith lattice can be written as

$$R_{i}=2l_{i}\sqrt{1-{\left(\psi_{i}\bar{H}\right)}^{2}},$$

the restriction R_i_ ≤ slantR_i+1_ is equivalent to

4.9 $$l_{i}\sqrt{1-{\left(\psi_{i}\bar{H}\right)}^{2}}⩽l_{i+1}\sqrt{1-{\left(\psi_{i+1}\bar{H}\right)}^{2}},$$

for i = 1, …, I − 1. This is equivalent to

4.10*a* $$\begin{array}{rl} & {\bar{H}}^{2}⩾\frac{l_{i}^{2}-l_{i+1}^{2}}{\psi_{i}^{2}l_{i}^{2}-\psi_{i+1}^{2}l_{i+1}^{2}}\;\mathrm{if}\;\frac{N_{i}}{M_{i}}>\frac{N_{i+1}}{M_{i+1}}, \end{array}$$

4.10*b* $$\begin{array}{rl} & l_{i}⩽l_{i+1}\;\;\;\;\;\mathrm{if}\;\frac{N_{i}}{M_{i}}=\frac{N_{i+1}}{M_{i+1}} \end{array}$$

4.10*c* $$\begin{array}{rl} \;\mathrm{and}\; & {\bar{H}}^{2}⩽\frac{l_{i}^{2}-l_{i+1}^{2}}{\psi_{i}^{2}l_{i}^{2}-\psi_{i+1}^{2}l_{i+1}^{2}}\;\mathrm{if}\;\frac{N_{i}}{M_{i}}<\frac{N_{i+1}}{M_{i+1}}, \end{array}$$

where we have used (4.1*a*) and (4.2*c*). Enforcing (4.10) for i = 1, …, I − 1, we obtain the range (which might be empty) of $\bar{H}$ for which radial self-intersections do not occur to be

4.11 $$\left[\sqrt{\max\left(0,\min_{\begin{array}{c} i\\ \left(N_{i}/M_{i})>(N_{i+1}/M_{i+1}\right) \end{array}}\frac{l_{i}^{2}-l_{i+1}^{2}}{\psi_{i}^{2}l_{i}^{2}-\psi_{i+1}^{2}l_{i+1}^{2}}\right)},\sqrt{\min\left(1,\max_{\begin{array}{c} i\\ \left(N_{i}/M_{i})<(N_{i+1}/M_{i+1}\right) \end{array}}\frac{l_{i}^{2}-l_{i+1}^{2}}{\psi_{i}^{2}l_{i}^{2}-\psi_{i+1}^{2}l_{i+1}^{2}}\right)}\right],$$

if l_i_ ≤ slantl_i+1_ for i = 1, …, I − 1; and empty otherwise. (Here we have taken into account that the range of $\bar{H}$ is [0, 1].)

#### (i) Absence of radial self-intersections

Radial self-intersections do not occur when

$$l_{i+1}>l_{i}\;\mathrm{and}\;\frac{N_{i}}{M_{i}}>\frac{N_{i+1}}{M_{i+1}},$$

for all i = 1, …, I − 1, since under these conditions the inner maximum on the right of (4.11) is vacuous, and the inner minimum on the left is non-positive. By a similar reasoning radial self-intersections do not occur also when

$$l_{i+1}<l_{i}\;\mathrm{and}\;\frac{N_{i}}{M_{i}}<\frac{N_{i+1}}{M_{i+1}},$$

for all i = 1, …, I − 1.

More comprehensively, (4.11) enables a characterization of the conditions on (l_i+1_/l_i_), (M_i+1_/M_i_) and (N_i+1_/N_i_) under which radial self-intersections do not occur. This would allow optimization to be conducted in two stages: first, the continuous design parameters for achieving the desired response can be obtained, assuming maximal range of motion. Second, banding numbers N_i_, M_i_ and unit cell lengths l_i_ are chosen to avoid radial self-intersections. See §8 for examples.

## 5. Taylor expansion of the system energy

In this section, we derive the Taylor expansions of the system energy (4.5) and present related results that are used in §6.

We denote the non-negative integers 0, 1, 2, … by $Z^{*}$, the L^∞^-norm of a continuous function f:[0,1]→R by ∥f∥_∞_:

$$∥f{∥}_{\infty }=\max_{x\in [0,1]}|f(x)|,$$

and the smallest/largest singular value of a square matrix T by *σ*_±_(T).

### (a) Taylor expansions of f_*ψ*_ and g_*ψ*_

Let *ψ*∈(0, 1], cf. (4.2*c*). Note that the maximal domain of f_*ψ*_ and g_*ψ*_, defined in (4.7), is ( − (1/*ψ*), (1/*ψ*)), which strictly contains [0, 1]. Thus, f_*ψ*_, g_*ψ*_∈C^∞^([0, 1]) are smooth inside and on the boundary of [0, 1]; indeed, they have Taylor expansions about 0 given by

4.7*a* $$\begin{array}{r} f_{\psi }(\bar{H})=\sqrt{1-{\left(\psi \bar{H}\right)}^{2}} \end{array}$$

5.1*a* =∑j=0∞(−1)j(12j)(ψH¯)2j=∑j=0∞c2j(ψH¯)2j

4.7*b* $$\begin{array}{r} \;\text{and}\;g_{\psi }(\bar{H})=\psi \bar{H}\sqrt{1-{\left(\psi \bar{H}\right)}^{2}} \end{array}$$

5.1*b* =∑j=0∞(−1)j(12j)(ψH¯)2j+1=∑j=0∞c2j+1(ψH¯)2j+1,

for $\bar{H}\in [0,1]$, cf. (4.2*d*). Here, for conciseness, we have set

5.2*a* c2j=c2j+1=(−1)j(12j),

for $j\in Z^{*}$. It is convenient to also define, for $J\in Z^{*}$,

5.2*b* $$\begin{array}{rl} C_{2J} & =\left(\begin{array}{cccc} c_{0} & 0 & \ldots & 0\\ 0 & c_{2} & \ldots & 0\\ \vdots & \vdots & & \vdots \\ 0 & 0 & \ldots & c_{2J} \end{array}\right)\in R^{\left(J+1)\times (J+1\right)} \end{array}$$

5.2*c* $$\begin{array}{rl} \text{and}\;C_{2J+1} & =\left(\begin{array}{cccc} c_{1} & 0 & \ldots & 0\\ 0 & c_{3} & \ldots & 0\\ \vdots & \vdots & & \vdots \\ 0 & 0 & \ldots & c_{2J+1} \end{array}\right)\in R^{\left(J+1)\times (J+1\right)}. \end{array}$$

We also list some properties of c_2j_, c_2j+1_, C_2J_ and C_2J+1_ which will be needed later:

**Remark 5.1.**

(i) |c_2j_| (and thus |c_2j+1_|) is a decreasing function of j.
(ii) C_2J_ and C_2J+1_ are invertible.
(iii) The smallest singular value of C_2J_ (and of C_2J+1_) is |c_2J_| = |c_2J+1_|.

**Proof.**

(i) We calculate
  $$\frac{|c_{2(j+1)}|}{|c_{2j}|}=\frac{|c_{2(j+1)+1}|}{|c_{2j+1}|}=\frac{1}{j+1}\left|j-\frac{1}{2}\right|<1.$$
(ii) This follows from c_2j_, c_2j+1_≠0 for $j\in Z^{*}$.
(iii) This is immediate from item (i) above. ▪

### (b) Truncated Taylor expansions of f_*ψ*_ and g_*ψ*_

Returning to (5.1), the finite truncations of the Taylor expansions of f_*ψ*_ and g_*ψ*_ are given by

5.3*a* $$\begin{array}{rl} P_{2J}(\psi \bar{H}) & =\sum_{j=0}^{J}c_{2j}{\left(\psi \bar{H}\right)}^{2j} \end{array}$$

5.3*b* $$\begin{array}{rl} \;\text{and}\;P_{2J+1}(\psi \bar{H}) & =\psi \bar{H}P_{2J}(\psi \bar{H})=\sum_{j=0}^{J}c_{2j+1}{\left(\psi \bar{H}\right)}^{2j+1}. \end{array}$$

Note that even though c_j_ < 0 unless j = 0, from (5.1), the polynomials above are positive for $\bar{H}\in [0,1]$.

By Taylor's theorem, we can truncate the expansions in (5.1). Given *ϵ* > 0, we can find $J_{P}(\epsilon )\in Z^{*}$ such that

5.4*a* $$f_{\psi }(\bar{H})=P_{2J_{P}}(\psi \bar{H})+R_{2J_{P}}(\psi \bar{H}),$$

where R_2J_P__(*ψ* · ), which is the remainder in the Taylor expansion of f_*ψ*_ to order 2J_P_, satisfies

5.4*b* $$∥R_{2J_{P}}(\psi \cdot ){∥}_{\infty }<\epsilon .$$

Then,

5.4*c* $$g_{\psi }(\bar{H})=P_{2J_{P}+1}(\psi \bar{H})+R_{2J_{P}+1}(\psi \bar{H}),$$

where

5.4*d* $$R_{2J_{P}+1}(\psi \bar{H})=\psi \bar{H}R_{2J_{P}}(\psi \bar{H}).$$

Since *ψ*∈(0, 1] and $\bar{H}\in [0,1]$, it follows that the remainder R_2J_P_ + 1_(*ψ* · ) also satisfies

5.4*e* $$∥R_{2J_{P}+1}(\psi \cdot ){∥}_{\infty }<\epsilon .$$

**Remark 5.2.**

J_P_ in (5.4) can be chosen so that the error, when (5.1) is truncated, is bounded by *ϵ*, independent of *ψ*. To see this, note that the remainder terms in (5.4*a,c*) are continuous for $(\psi ,\bar{H})\in {\left[0,1\right]}^{2}$, which is a compact set. (Here we have extended the domain of *ψ* to include 0 so as to obtain compactness.) Thus, to obtain bounds uniform in *ψ*, one chooses J_p_ to satisfy

5.5*a* $$\underset{\psi \in (0,1]}{\sup}∥R_{2J_{P}}(\psi \cdot ){∥}_{\infty }<\epsilon ,$$

instead of (5.4*b*). This implies

5.5*b* $$\underset{\psi \in (0,1]}{\sup}∥R_{2J_{P}+1}(\psi \cdot ){∥}_{\infty }<\epsilon ,$$

which is the uniform version of (5.4*e*).

In §5d, we analyse the system energy using the expansions and bounds above. However, before we do that, we present some properties of the Taylor polynomials in (5.3) that will be required there.

### (c) A basis for polynomials

Our first result is that the Taylor polynomials in (5.3) form a basis for the polynomials:

**Lemma 5.3.**

*Let* $J\in Z^{*}$ *and let* *ψ*_i_, *i* = 1, …, *J* + 1 *be distinct*. *Then*

(i) *The* *J* + 1 *polynomials P*_2*J*_(*ψ*_i_ · ), *i* = 1, …, *J* + 1, *defined in* (*5.3a*) *form a basis for the vector space of even polynomials of order at most* 2*J*.
(ii) *The J* + 1 *polynomials P*_2*J*+1_(*ψ*_i_ · ), *i* = 1, …, *J* + 1, *defined in* (*5.3b*) *form a basis for the vector space of odd polynomials of order at most* 2*J* + 1.

To prove the lemma, it is convenient to set, for I∈N,

5.6*a* $$W(\psi_{1},\psi_{2},\ldots ,\psi_{I})=\left(\begin{array}{cccc} \psi_{1} & 0 & \ldots & 0\\ 0 & \psi_{2} & \ldots & 0\\ \vdots & \vdots & & \vdots \\ 0 & 0 & \ldots & \psi_{I} \end{array}\right)\in R^{I\times I},$$

which is invertible when *ψ*_i_≠0, i = 1, …, I; and

5.6*b* $$V_{J}(\psi_{1},\psi_{2},\ldots ,\psi_{I})=\left(\begin{array}{cccc} 1 & \psi_{1}^{2} & \ldots & \psi_{1}^{2J}\\ 1 & \psi_{2}^{2} & \ldots & \psi_{2}^{2J}\\ \vdots & \vdots & & \vdots \\ 1 & \psi_{I}^{2} & \ldots & \psi_{I}^{2J} \end{array}\right)\in R^{I\times (J+1)},$$

which is square iff I = J + 1. Then, it is a Vandermonde matrix and is invertible when the *ψ*_i_ are distinct (cf. e.g. [28]).

**Proof of Lemma 5.3.**

From (5.3),

5.7*a* $$\begin{array}{rl} \left(\begin{array}{c} P_{2J}(\psi_{1}x)\\ P_{2J}(\psi_{2}x)\\ \vdots \\ P_{2J}(\psi_{J+1}x) \end{array}\right) & =\left(\begin{array}{cccc} c_{0} & c_{2}\psi_{1}^{2} & \ldots & c_{2J}\psi_{1}^{2J}\\ c_{0} & c_{2}\psi_{2}^{2} & \ldots & c_{2J}\psi_{2}^{2J}\\ \vdots & \vdots & & \vdots \\ c_{0} & c_{2}\psi_{J+1}^{2} & \ldots & c_{2J}\psi_{J+1}^{2J} \end{array}\right)\left(\begin{array}{c} 1\\ x^{2}\\ \vdots \\ x^{2J} \end{array}\right)\\ & =V_{J}(\psi_{1},\psi_{2},\ldots ,\psi_{J+1})\;C_{2J}\left(\begin{array}{c} 1\\ x^{2}\\ \vdots \\ x^{2J} \end{array}\right). \end{array}$$

Now, the polynomials x^2j^, j = 0, …, J, form a basis for the vector space of even polynomials of order at most 2J. Since both matrices in (5.7*a*) are invertible it follows that the same is true for the polynomials P_2J_(*ψ*_i_ · ), i = 1, …, J + 1.

The proof of the second assertion for odd polynomials is similar and follows from:

5.7*b* $$\begin{array}{rl} \left(\begin{array}{c} P_{2J+1}(\psi_{1}x)\\ P_{2J+1}(\psi_{2}x)\\ \vdots \\ P_{2J+1}(\psi_{J+1}x) \end{array}\right) & =\left(\begin{array}{cccc} c_{1}\psi_{1} & c_{3}\psi_{1}^{3} & \ldots & c_{2J+1}\psi_{1}^{2J+1}\\ c_{1}\psi_{2} & c_{3}\psi_{2}^{3} & \ldots & c_{2J+1}\psi_{2}^{2J+1}\\ \vdots & \vdots & & \vdots \\ c_{1}\psi_{J+1} & c_{3}\psi_{J+1}^{3} & \ldots & c_{2J+1}\psi_{J+1}^{2J+1} \end{array}\right)\left(\begin{array}{c} x\\ x^{3}\\ \vdots \\ x^{2J+1} \end{array}\right)\\ & =W(\psi_{1},\psi_{2},\ldots ,\psi_{J+1})\;V_{J}(\psi_{1},\psi_{2},\ldots ,\psi_{J+1})\;C_{2J+1}\left(\begin{array}{c} x\\ x^{3}\\ \vdots \\ x^{2J+1} \end{array}\right), \end{array}$$

and the invertibility of the matrices in (5.7*b*). ▪

Combining the two assertions of lemma 5.3, we immediately obtain:

**Corollary 5.4.**

*Let* *ψ*_1_, *ψ*_2_, …, *ψ*_*J*+1_∈(0, 1] *be distinct*. *Then*, *the* 2*J* + 2 *polynomials*

$$\bar{H}\mapsto P_{2J}(\psi_{i}\bar{H}),\;\bar{H}\mapsto P_{2J+1}(\psi_{i}\bar{H}),\;i=1,\ldots ,J+1,$$

*presented in* (*5.3*)*, form a basis for the vector space of polynomials of order at most* 2*J* + 1.

We shall refer to the basis in corollary 5.4 as the P-basis.

As our final result for this section, we present, for later use, a bound on the singular values of the square Vandermonde matrix V _J_(*ψ*_1_, *ψ*_2_, …, *ψ*_J+1_) defined in (5.6*b*). This immediately follows, for the case *ψ*_1_, *ψ*_2_, …, *ψ*_J+1_∈(0, 1], from the result in [29, section 3.2].

**Lemma 5.5.**

*Let* *ψ*_1_, *ψ*_2_, …, *ψ*_*J*+1_∈(0, 1] *be distinct*. *Then*,

5.8 $$\sigma_{-}⩾\frac{1}{\sqrt{J+1}}\max_{1⩽k<J+1}\prod_{\begin{array}{c} \ell =1,\\ \ell \neq k \end{array}}^{J+1}\frac{1}{|\psi_{k}^{2}-\psi_{\ell }^{2}|},$$

*where* *σ*_−_ *is the smallest singular value of V*_*N*_(*ψ*_1_, *ψ*_2_, …, *ψ*_*J*+1_).

### (d) Truncated Taylor expansion of the system energy

From the Taylor expansions in (5.4), given $J\in Z^{*}$, we have a Taylor expansion for (4.5):

4.5 $$\begin{array}{rl} \Xi (\bar{H}) & =A_{0}+A_{1}\bar{H}+A_{2}{\bar{H}}^{2}+\sum_{i=1}^{I}B_{i}\;f_{\psi_{i}}(\bar{H})+\sum_{i=1}^{I}C_{i}\;g_{\psi_{i}}(\bar{H}) \end{array}$$

5.9*a* $$\begin{array}{r} \approx A_{0}+A_{1}\bar{H}+A_{2}{\bar{H}}^{2}+\sum_{i=1}^{I}B_{i}\;P_{2J}(\psi_{i}\bar{H})+\sum_{i=1}^{I}C_{i}\;P_{2J+1}(\psi_{i}\bar{H}) \end{array}$$

5.9*b* $$\begin{array}{rl} & =\left(A_{0}+c_{0}\sum_{i=1}^{I}B_{i}\right)+\left(A_{1}+c_{1}\sum_{i=1}^{I}C_{i}\;\psi_{i}\right)\bar{H}+\left(A_{2}+c_{2}\sum_{i=1}^{I}B_{i}\;\psi_{i}^{2}\right){\bar{H}}^{2}\\ & \;+c_{3}\left(\sum_{i=1}^{I}C_{i}\;\psi_{i}^{3}\right){\bar{H}}^{3}+c_{4}\left(\sum_{i=1}^{I}B_{i}\;\psi_{i}^{4}\right){\bar{H}}^{4}\\ & \;+\ldots +c_{2J}\left(\sum_{i=1}^{I}B_{i}\;\psi_{i}^{2J}\right){\bar{H}}^{2J}+c_{2J+1}\left(\sum_{i=1}^{I}C_{i}\;\psi_{i}^{2J+1}\right){\bar{H}}^{2J+1}. \end{array}$$

The terms of order 0, 1 and 2 have contributions from A_0_, A_1_ and A_2_, respectively, but the higher-order odd and even terms follow the pattern of the terms of order 3 and 4, respectively.

From (5.4), the L^∞^-error in (5.9*a*) is

5.10*a* $$\begin{array}{rl} {∥\sum_{i=1}^{I}B_{i}\;R_{2J}(\psi_{i}\bar{H})+\sum_{i=1}^{I}C_{i}\;R_{2J+1}(\psi_{i}\bar{H})∥}_{\infty } & ⩽IB_{+}∥R_{2J}{∥}_{\infty }+IC_{+}∥R_{2J+1}{∥}_{\infty }\\ & ⩽2I\max\left(B_{+},C_{+}\right)\;\max\left(∥R_{2J}{∥}_{\infty },∥R_{2J+1}{∥}_{\infty }\right), \end{array}$$

5.10*b* $$\begin{array}{rl} \;\text{where}\;B_{+} & =\max_{i=1,\ldots ,I}|B_{i}| \end{array}$$

5.10*c* $$\begin{array}{rl} \;\text{and}\;C_{+} & =\max_{i=1,\ldots ,I}|C_{i}|. \end{array}$$

As our last result for this section, we write the Taylor expansion (5.9) in the P-basis (cf. corollary 5.4 and (5.3)).

For the even coefficients of the polynomial in (5.9*b*), using (5.6), we obtain,

5.11*a* $$\left(\begin{array}{c} A_{0}+c_{0}\sum_{i=1}^{I}B_{i}\\ A_{2}+c_{2}\sum_{i=1}^{I}B_{i}\;\psi_{i}^{2}\\ c_{4}\sum_{i=1}^{I}B_{i}\;\psi_{i}^{4}\\ \vdots \\ c_{2J}\sum_{i=1}^{I}B_{i}\;\psi_{i}^{2J} \end{array}\right)=\left(\begin{array}{c} A_{0}\\ A_{2}\\ 0\\ \vdots \\ 0 \end{array}\right)+C_{2J}\;V_{2J}(\psi_{1},\psi_{2},\ldots ,\psi_{I})\left(\begin{array}{c} B_{1}\\ B_{2}\\ B_{3}\\ \vdots \\ B_{I} \end{array}\right).$$

Similarly, for the odd coefficients of the polynomial in (5.9*b*) we have

5.11*b* $$\left(\begin{array}{c} A_{1}+c_{1}\sum_{i=1}^{I}C_{i}\;\psi_{i}\\ c_{3}\sum_{i=1}^{I}C_{i}\;\psi_{i}^{3}\\ \vdots \\ c_{2J+1}\sum_{i=1}^{I}C_{i}\;\psi_{i}^{2J+1} \end{array}\right)=\left(\begin{array}{c} A_{1}\\ 0\\ \vdots \\ 0 \end{array}\right)+C_{2J+1}\;W(\psi_{1},\psi_{2},\ldots ,\psi_{I})\;V_{2J}(\psi_{1},\psi_{2},\ldots ,\psi_{I})\left(\begin{array}{c} C_{1}\\ C_{2}\\ \vdots \\ C_{I} \end{array}\right).$$

## 6. Lattice systems approximating a prescribed energy functional

In this section, we prove our main result, namely, that given *ϵ* > 0 and an energy E∈C([0,1],R), a lattice system can be constructed whose energy, is approximately E up to an additive constant, with L^∞^-error no more than *ϵ*.

In fact, it suffices to show this for polynomial energies since, from Weierstrass approximation theorem [30], we can find a polynomial $E^{P}:[0,1]\to R$ such that

6.1*a* $$\max_{\bar{H}\in [0,1]}\left|E(\bar{H})-E^{P}(\bar{H})\right|<\frac{\epsilon }{2}.$$

In the light of this, we will show that, given *ϵ* > 0 and a polynomial energy $E^{P}:[0,1]\to R$, a lattice system can be constructed whose energy, *Ξ*, is approximately E^P^, up to an additive constant, E_0_, with L^∞^-error no more than (*ϵ*/2):

6.1*b* $$\max_{\bar{H}\in [0,1]}\left|E^{P}(\bar{H})-\Xi (\bar{H})-E_{0}\right|<\frac{\epsilon }{2}.$$

Then, it would follow from (6.1) and the triangle inequality that

$$\max_{\bar{H}\in [0,1]}\left|E(\bar{H})-\Xi (\bar{H})-E_{0}\right|<\max_{\bar{H}\in [0,1]}\left|E(\bar{H})-E^{P}(\bar{H})\right|+\max_{\bar{H}\in [0,1]}\left|E^{P}(\bar{H})-\Xi (\bar{H})-E_{0}\right|<\epsilon .$$

We will prove this assertion in two steps: First, we will show in theorem 6.2 that, given a polynomial energy $E^{P}:[0,1]\to R$, of the form

6.2 $$E^{P}(\bar{H})=e_{0}+e_{1}\bar{H}+e_{2}{\bar{H}}^{2}+e_{3}{\bar{H}}^{3}+\ldots +e_{2N+1}{\bar{H}}^{2N+1},$$

for some $N\in Z^{*}$, there exists an appropriate choice of lattice system parameters such that the system energy (4.5) differs from E^P^ by no more than (*ϵ*/2) in L^∞^. Then, we will show in theorem 6.3 that lattice systems with these parameters, with the possible exception of the constant term A_0_ in (4.5), can actually be constructed.

**Remark 6.1.**

For convenience, we will allow for the possibility that e_2N+1_ = 0 in (6.2). This allows us to assume, with no loss of generality, that the highest power of the polynomial E^P^ is odd.

### (a) Approximation of polynomial energies

**Theorem 6.2**

*Given* *ϵ* > 0 *and a polynomial* E^P^, *there exist*

6.3*a* $$\begin{array}{rl} I & \in N, \end{array}$$

6.3*b* $$\begin{array}{rl} \psi_{i} & \in (0,1],\;i=1,\ldots ,I, \end{array}$$

6.3*c* $$\begin{array}{rl} A_{0},A_{1},A_{2} & \in R \end{array}$$

6.3*d* $$\begin{array}{rl} \;\mathrm{and}\;B_{i},C_{i} & \in R,\;i=1,\ldots ,I, \end{array}$$

*such that the lattice system energy* (*4.5*) *differs from* E^P^ *by no more than* (*ϵ*/2) *in* L^∞^.

**Proof.**

From remark 6.1, we may assume that the highest power of $\bar{H}$ in E^P^ is 2N + 1, for some $N\in Z^{*}$. Set I = N + 1 and pick D > 0 such that

6.4 $$\max\left(\underset{\psi \in (0,1]}{\sup}∥R_{2N_{P}}(\psi \cdot ){∥}_{\infty },\underset{\psi \in (0,1]}{\sup}∥R_{2N_{P}+1}(\psi \cdot ){∥}_{\infty }\right)⩽\frac{\epsilon }{4ID},$$

where R_2N_P__ and R_2N_P_ + 1_ are defined in (5.4*a,c*); this is possible by remark 5.2.

Now suppose *ψ*_i_∈(0, 1], i = 1, …, I are distinct. Then, we can write the polynomial E^P^ in the P-basis (cf. corollary 5.4 and subsequent remarks), i.e. P_2N_(*ψ*_i_ · ), P_2N+1_(*ψ*_i_ · ), i = 1, …, I, as in (5.3); indeed, we can write it as a (non-unique) combination of P- and monomial-bases. To do so, we would find (non-unique) solutions to the affine equations

6.5*a* $$\begin{array}{rl} \left(\begin{array}{c} e_{0}\\ e_{2}\\ \vdots \\ e_{2N} \end{array}\right) & =\left(\begin{array}{c} A_{0}\\ A_{2}\\ 0\\ \vdots \\ 0 \end{array}\right)+C_{2N}\;V_{N}(\psi_{1},\psi_{2},\ldots ,\psi_{I})\left(\begin{array}{c} B_{1}\\ B_{2}\\ B_{3}\\ \vdots \\ B_{I} \end{array}\right) \end{array}$$

6.5*b* $$\begin{array}{rl} \;\text{and}\;\left(\begin{array}{c} e_{1}\\ e_{3}\\ \vdots \\ e_{2N+1} \end{array}\right) & =\left(\begin{array}{c} A_{1}\\ 0\\ \vdots \\ 0 \end{array}\right)+C_{2N+1}\;W(\psi_{1},\psi_{2},\ldots ,\psi_{I})\;V_{N}(\psi_{1},\psi_{2},\ldots ,\psi_{I})\left(\begin{array}{c} C_{1}\\ C_{2}\\ \vdots \\ C_{I} \end{array}\right). \end{array}$$

The existence of solutions to these equations follows from the invertibility of the four matrices in (6.5), see remark 5.1(ii) and remarks following (5.6). The solution space is three-dimensional as can be seen by observing that solutions exist for any A_0_, A_1_, A_2_, and are unique once these are fixed (again, by invertibility of the four matrices that appear in (6.5)).

Now choose A_j_, j = 1, 2, 3, and let

$$\begin{array}{rl} E_{e} & =\max\left(|e_{0}-A_{0}|,|e_{2}-A_{2}|,|e_{4}|,\ldots ,|e_{2N}|\right)\\ \;\text{and}\;E_{o} & =\max\left(|e_{1}-A_{1}|,|e_{3}|,\ldots ,|e_{2N+1}|\right). \end{array}$$

We wish to solve (6.5) under the constraint that

6.6 $$\max_{i=1,\ldots ,I}\max\left(B_{i},C_{i}\right)⩽D$$

(cf. (5.10) and (6.4)). In other words, the image of a ball of radius D under the linear map C_2N_ V _N_(*ψ*_1_, *ψ*_2_, …, *ψ*_I_) needs to contain a ball of radius E_e_; and the image of a ball of radius D under the linear map C_2N+1_ W(*ψ*_1_, *ψ*_2_, …, *ψ*_I_) V _N_(*ψ*_1_, *ψ*_2_, …, *ψ*_I_) needs to contain a ball of radius E_o_. This is equivalent to the requirement that

$$\begin{array}{rl} \sigma_{-}(C_{2N}\;V_{N}(\psi_{1},\psi_{2},\ldots ,\psi_{I})) & ⩾\frac{E_{e}}{D}\\ \;\text{and}\;\sigma_{-}(C_{2N+1}\;W(\psi_{1},\psi_{2},\ldots ,\psi_{I})\;V_{N}(\psi_{1},\psi_{2},\ldots ,\psi_{I})) & ⩾\frac{E_{o}}{D}. \end{array}$$

From (5.2*a*), remark 5.1(iii), (5.6*a*) and (5.8), a sufficient condition for this is

6.7*a* 1N+1 |(12N)| max1⩽k<N+1∏ℓ=1,ℓ≠kN+11|ψk2−ψℓ2|⩾EeD

6.7*b* and1N+1 |(12N)| (mini=1,…,Iψi) (max1⩽k<N+1∏ℓ=1,ℓ≠kN+11|ψk2−ψℓ2|)⩾EoD.

Now choose *ψ*_i_∈(0, 1], i = 1, …, I, distinct so as to satisfy (6.7); this is possible since the expressions on the left in (6.7) can be made arbitrarily large by choosing as many *ψ*_i_s as necessary to be sufficiently close to each other.

Finally, let B_i_, C_i_, i = 1, …, I solve (6.5). By the construction above (6.6) is satisfied.

The lattice system with the parameters chosen above has energy given by (4.5). By (5.9*a*,*b*) and (5.11) and (6.6), the Taylor expansion of this energy to order 2N + 1 is precisely the given polynomial E^P^. From (5.10), (6.4) and (6.6) the error introduced by the Taylor approximation is bounded by (*ϵ*/2). ▪

### (b) Existence of lattice systems with required energy

Next we show, in theorem 6.3, that a lattice system can be constructed that attains the energy in theorem 6.2, up to an additive constant. We do this by showing that, for each lattice in the system, the material parameters ${\bar{\delta }}_{\pm },{\bar{\epsilon }}_{\pm },{\bar{\upsilon }}_{x\pm },{\bar{\upsilon }}_{xy\pm },\bar{\varphi }$ and the geometric-material parameter *Υ* can be chosen so as to yield the coefficients (6.3) required by theorem 6.2, except possibly for A_0_. In fact, we shall show that this is possible even when we impose the additional constraints

6.8*a* $$\begin{array}{rl} {\bar{\delta }}_{+} & ={\bar{\delta }}_{-}, \end{array}$$

6.8*b* $$\begin{array}{rl} {\bar{\epsilon }}_{+} & ={\bar{\epsilon }}_{-}, \end{array}$$

6.8*c* $$\begin{array}{rl} {\bar{\upsilon }}_{x+} & ={\bar{\upsilon }}_{x-} \end{array}$$

6.8*d* $$\begin{array}{rl} \mathrm{and}\;{\bar{\upsilon }}_{xy+} & ={\bar{\upsilon }}_{xy-}, \end{array}$$

on each lattice in the system.

**Theorem 6.3.**

*Given the coefficients in* (*6.3*), *i*.*e*. *given*

$$\begin{array}{rl} & I\in N,\\ & \psi_{i}\in (0,1],\;i=1,\ldots ,I,\\ & A_{1},A_{2}\in R\\ \;\mathrm{and}\; & B_{i},C_{i}\in R,\;i=1,\ldots ,I, \end{array}$$

*there exist* ${\bar{\delta }}_{i\pm },$ ${\bar{\epsilon }}_{i\pm },$ ${\bar{\upsilon }}_{ix\pm },$ ${\bar{\upsilon }}_{ixy\pm },$ ${\bar{\varphi }}_{i},$ $\Upsilon_{i}\in R,$ i = 1, …, I, *satisfying* (*3.9*)*, such that* (*3.8b–e*) *and* (*4.4b*) *are satisfied*.

Note that A_0_ is omitted from the list of coefficients in (6.3) since the energy of the lattice system in theorem 6.3 is allowed to differ from the energy of the lattice system in theorem 6.2 by an additive constant. Similarly, in the proof below we will omit (3.8*a*) from the (3.8).

**Proof.**

Pick *Υ*_i_ > 0, i = 1, …, I. It is convenient to view this as a choice of $\underline{\Upsilon }\in R^{I}$ with positive components. Notice that (4.6*b–e*) can be written as

6.9*a* $$\begin{array}{rl} W(\psi_{1},\psi_{2},\ldots ,\psi_{I})\underline{\Upsilon }\cdot \underline{a_{1}} & =A_{1}, \end{array}$$

6.9*b* $$\begin{array}{rl} W{\left(\psi_{1},\psi_{2},\ldots ,\psi_{I}\right)}^{2}\underline{\Upsilon }\cdot \underline{a_{2}} & =A_{2}, \end{array}$$

6.9*c* $$\begin{array}{rl} W(\Upsilon_{1},\Upsilon_{2},\ldots ,\Upsilon_{I})\underline{b_{1}} & =\underline{B} \end{array}$$

6.9*d* $$\begin{array}{rl} \text{and}\;W(\Upsilon_{1},\Upsilon_{2},\ldots ,\Upsilon_{I})\underline{b_{2}} & =\underline{C}, \end{array}$$

where $\underline{a_{1}},$ $\underline{a_{2}},$ $\underline{B},$ $\underline{C}\in R^{I}$.

Here, W, defined in (5.6*a*), is invertible since *Υ*_i_ > 0, i = 1, …, I. Thus, (6.9*c*) and (6.9*d*) yield $\underline{b_{1}}$ and $\underline{b_{2}}$, respectively. Each of (6.9*a*) and (6.9*b*) has an (I − 1)-dimensional space of solutions since these equations define affine planes in $R^{I}$ with co-dimension 1. Thus, we have a choice of coefficients a_1i_, a_2i_, b_1i_, $b_{2i}\in R$, i = 1, …, I, which satisfy (4.6*b–e*).

Next, for each lattice, given the coefficients a_1_, a_2_, b_1_, $b_{2}\in R$ we shall construct a solution to (3.8*b*–*e*), which satisfies (3.9).

To do this, we first pick $\bar{\varphi }\in R$ to satisfy

6.10*a* $$\begin{array}{r} \bar{\varphi }>\max\left(0,-1-a_{2}\right) \end{array}$$

6.10*b* $$\begin{array}{r} \;\text{and}\;{\bar{\varphi }}^{3}+(a_{2}-1){\bar{\varphi }}^{2}-\left(1+2a_{2}+\frac{b_{2}^{2}}{4}\right)\bar{\varphi }+\left(1+a_{2}-\frac{b_{2}^{2}}{4}\right)>0. \end{array}$$

This is possible since the coefficient of the highest power of the polynomial above is positive, guaranteeing that a sufficiently large $\bar{\varphi }$ will satisfy both inequalities. Note that (6.10*a*) implies (3.9*a*).

Next we pick

$${\bar{\delta }}_{+}(\bar{\varphi })={\bar{\delta }}_{-}(\bar{\varphi })=1+\frac{a_{2}}{1+\bar{\varphi }}\;\mathrm{and}\;{\bar{\epsilon }}_{+}(\bar{\varphi })={\bar{\epsilon }}_{-}(\bar{\varphi })=\frac{b_{2}}{2(1-\bar{\varphi })}.$$

It is easy to see that (3.8*c,e*) are satisfied. Moreover, from (6.10*a*), (3.9*b*) is also satisfied.

We now calculate

$${\bar{\epsilon }}_{+}^{2}-{\bar{\delta }}_{+}={\bar{\epsilon }}_{-}^{2}-{\bar{\delta }}_{-}=\frac{b_{2}^{2}}{4{\left(1-\bar{\varphi }\right)}^{2}}-\left(1+\frac{a_{2}}{1+\bar{\varphi }}\right),$$

and an algebraic calculation shows that this is negative precisely when (6.10*b*) is satisfied; thus (3.9*c*) is satisfied.

Finally, we pick ${\bar{\upsilon }}_{x\pm }$ and ${\bar{\upsilon }}_{xy\pm }$ to satisfy

$$\left(\begin{array}{cc} 1 & \bar{\epsilon }\\ \bar{\epsilon } & \bar{\delta } \end{array}\right)\left(\begin{array}{c} {\bar{\upsilon }}_{x\pm }\\ {\bar{\upsilon }}_{xy\pm } \end{array}\right)=-\frac{1}{2}\left(\begin{array}{c} \frac{b_{1}}{1+\bar{\varphi }}\\ \frac{a_{1}}{1-\bar{\varphi }} \end{array}\right),$$

which is simply (3.8*b,d*) written as a linear system. This has a unique solution since, by (3.9*c*), the matrix is invertible. This completes the proof. ▪

Note the considerable freedom in the choice of coefficients and geometric and material parameters in the constructions in the proofs of theorems 6.2 and 6.3. In particular, as the computational examples in §8 show, the lattices in the system need not satisfy (6.8).

## 7. Ancillary results

### (a) Simplification of the choice of *ψ*_i_∈(0, 1], i = 1, …, I

The choice of (distinct) *ψ*_i_∈(0, 1], i = 1, …, I, so as to satisfy (6.7) can be simplified by the following two remarks:

**Remark 7.1.**

The bounds in (6.7) can be simplified (but made less tight and therefore more restrictive) using the observation that

$$\prod_{\begin{array}{c} \ell =1,\\ \ell \neq k \end{array}}^{N+1}\frac{1}{|\psi_{k}^{2}-\psi_{\ell }^{2}|}=\left(\prod_{\begin{array}{c} \ell =1,\\ \ell \neq k \end{array}}^{N+1}\frac{1}{|\psi_{k}+\psi_{\ell }|}\right)\left(\prod_{\begin{array}{c} \ell =1,\\ \ell \neq k \end{array}}^{N+1}\frac{1}{|\psi_{k}-\psi_{\ell }|}\right)>\frac{1}{2^{N}}\prod_{\begin{array}{c} \ell =1,\\ \ell \neq k \end{array}}^{N+1}\frac{1}{|\psi_{k}-\psi_{\ell }|}.$$

(This follows from |*ψ*_k_ + *ψ*_ℓ_| ≤ slant2 for ℓ = 1, …, N + 1.) Thus, to satisfy (6.7) it suffices to enforce:

7.1*a* max1⩽k<N+1∏ℓ=1,ℓ≠kN+11|ψk−ψℓ|⩾N+1 2N|((1/2)N)| EeD

7.1*b* and(mini=1,…,Iψi) (max1⩽k<N+1∏ℓ=1,ℓ≠kN+11|ψk−ψℓ|)⩾N+1 2N|((1/2)N)| EoD.

**Remark 7.2.**

(i) To satisfy (6.7), it suffices to enforce (6.7*b*) and
  7.2*a* $$\min_{i=1,\ldots ,I}\psi_{i}⩽\frac{E_{o}}{E_{e}}.$$
  Of course, (7.2*a*) is trivially satisfied if (E_o_/E_e_)≥1 so, in that case, (6.7) is equivalent to (6.7*b*).
(ii) On the other hand, when (E_o_/E_e_) < 1, to satisfy (6.7) it suffices to enforce (6.7*a*) and
  7.2*b* $$\min_{i=1,\ldots ,I}\psi_{i}⩾\frac{E_{o}}{E_{e}}.$$

Note that, in light of remarks 7.1 and 7.2 also holds when (6.7), (6.7*a*) and (6.7*b*) are replaced by (7.1), (7.1*a*) and (7.1*b*), respectively.

**Proof.**

(i) From (6.7*b*) and (7.2*a*), we obtain
  $$l.h.s.\;(6.7a)⩾\frac{1}{\min_{i=1,\ldots ,I}\psi_{i}}\;\frac{E_{o}}{D}⩾\frac{E_{e}}{D},$$
  which is (6.7*a*).
(ii) From (6.7*a*) and (7.2*b*), we obtain
  $$l.h.s.\;(6.7b)⩾\left(\min_{i=1,\ldots ,I}\psi_{i}\right)\frac{E_{o}}{D}\;⩾\frac{E_{e}}{D},$$
  which is (6.7*b*). ▪

### (b) Preservation of local extrema

While the construction presented in §6 guarantees a lattice system whose energy is close to a prescribed function, there is no restriction on how the system energy behaves within the tolerance band. In particular, the system energy could eliminate local extremizers of the prescribed energy. However, for many applications, it is desirable that the system energy retain these local extremizers.

We now show that this can be easily incorporated into our framework, provided that the number of local extremizers is finite.

Given an energy E∈C([0,1],R), let

$$\begin{array}{rl} m_{E} & =\{\bar{H}\in [0,1]\;|\;\bar{H}\text{is a local minimum of }\;E\}\\ \;\text{and}\;M_{E} & =\{\bar{H}\in [0,1]\;|\;\bar{H}\text{is a local maximum of }E\}, \end{array}$$

be the sets of local minimizers and maximizers of E, respectively. We assume that these sets are finite. For the system energy, *Ξ*, to be able to distinguish between adjacent local extremizers m∈m_E_ and M∈M_E_ we need the tolerance *ϵ* to satisfy

E(m)+ϵ<E(M)−ϵ.

In other words, we need

7.3 $$\epsilon <\frac{1}{2}\left(E(M)-E(m)\right),$$

for every pair of adjacent local extremizers m∈m_E_ and M∈M_E_. (Finiteness of m_E_ and M_E_ ensures that the minimum of l.h.s. (7.3) is positive.) To see the sufficiency of (7.3), consider adjacent local extrema m_−_ < M < m_+_ where m_−_ , m_+_∈m_E_ and M∈M_E_. Using (7.3), we have

$$\Xi (m_{\pm })⩽E(m_{\pm })+\epsilon <E(M)-\epsilon ⩽\Xi (M),$$

from which it follows that *Ξ* has a local maximum in (m_−_ , m_+_), although it need not be located at M. A similar result holds for adjacent M_−_ < m < M_+_ where m∈m_E_ and M_−_ , M_+_∈M_E_.

Thus, *Ξ* preserves the local extremizers of E in the sense that *Ξ* has a local minimizer/maximizer between any two adjacent local maximizer/minimizer of E, respectively. Note, however that it is possible that *Ξ* has more extremizers than E.

The following counterexample shows that the condition that E have only finitely many local extremizers is essential.

**Example 7.3.**

The energy

$$E(\bar{H})=\left\{ \begin{array}{ll} {\bar{H}}^{2}\sin\frac{1}{\bar{H}} & \mathrm{for}\;\bar{H}\in (0,1],\\ 0 & \mathrm{for}\;\bar{H}=0, \end{array}\right.$$

is continuous (in fact it is absolutely continuous and thus differentiable a.e., cf. e.g. [31, p. 179, 209]). However, it is clear that it has infinitely many local extremizers, only finitely many of which can be distinguished for any tolerance *ϵ* > 0.

### (c) Specification of local extremizers

It might be that, rather than approximating an energy, we are concerned only with approximating its extremizers. For example, we might desire that a system have local minimizers at specified values of $\bar{H}$, with the precise energy being unimportant. Suppose we want an energy with local minimizers at

7.4 $$0⩽m_{1}<m_{2}<\cdots <m_{K}⩽1,$$

for some integer K > 1. (The trivial case K = 1 is considered below.) Then, we pick M_1_, M_2_, …, M_K−1_ such that

$$m_{k}<M_{k}<m_{k+1},$$

for k = 1, …, K − 1, and set

7.5*a* $$F^{P}(\bar{H})=\left(\prod_{k=1}^{K}(\bar{H}-m_{k})\right)\left(\prod_{k=1}^{K-1}(\bar{H}-M_{k})\right),$$

so that the roots of F^P^ are precisely m_1_, M_1_, m_2_, M_2_, …, M_K−1_, m_K_. Now let

7.5*b* $$E^{P}(\bar{H})=E_{0}^{P}+\int_{0}^{\bar{H}}F^{P}(x)\;dx,$$

for some $E_{0}^{P}\in R$. Then, the local minimizers of E^P^ are precisely m_1_, m_2_, …, m_K_.

To see this, note first from (7.5) that m_1_, m_2_, …, m_K_ are extremizers of E^P^. From (7.5*a*),

7.6 $$F^{P}(0)={\left(-1\right)}^{2K-1}\left(\prod_{k=1}^{K}m_{k}\right)\left(\prod_{k=1}^{K-1}M_{k}\right)\;\left\{ \begin{array}{ll} =0 & \mathrm{if}\;m_{1}=0,\\ <0 & \mathrm{if}\;m_{1}>0. \end{array}\right.$$

On the other hand, for $\bar{H}\in (m_{1},M_{1})$, from (7.5*a*), $F^{P}(\bar{H})>0$ since it is the product of the positive factor $\bar{H}-m_{1}$ and 2(K − 1) negative factors. From this, we deduce that m_1_ is indeed a local minimizer of E^P^. It is clear from (7.5) that the same is true for m_2_, …, m_K_.

(When K = 1, we choose $F^{P}(\bar{H})=\bar{H}-m_{1};$ it is clear that the resulting E^P^ has a unique minimizer at m_1_.)

We can now approximate the polynomial energy E^P^ as per §6a,b. Note the K degrees of design freedom that results from the ability to choose M_k_, k = 1, …, K − 1 and E^P^_0_ in (7.5*b*).

From the reasoning above, it is clear that an energy with local maximizers at (7.4) can be obtained by changing the sign of (7.5*a*).

### (d) Approximating derivatives of the energy

In reality, one is rarely concerned with prescribing a system's energy. Instead, one typically desires to specify equilibria, reaction forces and stiffnesses. In other words, it is not quite the energy landscape but rather its first and second derivatives that are prescribed.

However, as the following simple example illustrates, the derivative of an approximate energy need not approximate the derivative of energy. Consider the energy

$$E(\bar{H})=\delta \sin\left(\frac{\bar{H}}{\delta }\right),$$

for some *δ* > 0, and suppose we wish to approximate it to error *ϵ* > *δ*.

Since

$$|E(\bar{H})|<\delta <\epsilon ,$$

the constant polynomial

$$E^{P}(\bar{H})=0,$$

approximates this energy to the desired accuracy. From theorems 6.2 and 6.3, we can construct a lattice system whose energy approximates E up to an additive constant. (In this case, the lattice ‘system’ is trivial since there is only one lattice, i.e. I = 1; see [18, Section 5]). So we have

$$\Xi (\bar{H})=E_{0},$$

for some constant E_0_.

Thus, the derivatives of both the polynomial approximation of the energy and the lattice energy are

$${E^{P}}^{\prime }(\bar{H})=\Xi^{\prime }(\bar{H})=0.$$

However,

$$E^{\prime }(\bar{H})=\cos\left(\frac{\bar{H}}{\delta }\right),$$

and, when *ϵ* > 1, it is not true that

$$\left|E^{\prime }(\bar{H})-\Xi^{\prime }(\bar{H})\right|<\epsilon ,$$

since

$$\left|E^{\prime }(0)-\Xi^{\prime }(0)\right|=1>\epsilon .$$

Thus, the derivative of an approximate energy need not approximate the true derivative of the energy.

Moreover, this is true even if we allow for different tolerances for the energy and its derivative. To see this consider the following slight modification of example 7.3:

**Example 7.4.**

The energy

$$E(\bar{H})=\left\{ \begin{array}{ll} \bar{H}\sin\frac{1}{\bar{H}} & \mathrm{for}\;\bar{H}\in (0,1],\\ 0 & \mathrm{for}\;\bar{H}=0, \end{array}\right.$$

is continuous in [0, 1]. Its derivative,

$$E^{\prime }(\bar{H})=\sin\left(\frac{1}{\bar{H}}\right)-\frac{1}{\bar{H}}\cos\left(\frac{1}{\bar{H}}\right),$$

exists in (0, 1], but not at 0. However, for any tolerance, no polynomial can approximate E′ in (0, 1] since

$$\underset{\bar{H}\to 0}{lim sup}E^{\prime }(\bar{H})=\infty .$$

Thus, in order to approximate the first or second derivative of the system energy one would have to repeat the analysis in §§5 and 6 for the expressions in (4.8), which analysis we shall present elsewhere. In the meantime, we remark that examples such as the two presented above are pathological and the derivatives of reasonable energies can indeed be approximated. We demonstrate this computationally in §8.

## 8. Examples of bespoke system design

We now proceed to demonstrate the ability to tailor the energy landscape by presenting four examples of bespoke nonlinear elastic responses designed as described above:

(i) A monostable system with specified nonlinear stiffness (Example 1 in §8c).
(ii) A bi-stable system with specified local minima (Example 2 in §8d).
(ii) A multi-stable system with specified local minima (Example 3 in §8e).
(iv) A multi-stable system with specified snap-through loads (Example 4 in §8f).

Although the analysis is valid for $\bar{H}\in [0,1]$, for the computational examples, we choose to limit the extension to $\bar{H}\in [0,0.8]$ for the following reason: Because the truncated Taylor expansions in §5b are less accurate when $\bar{H}\to 1$, the designer can account for this by *a priori* choosing to work with less than the maximum possible extension. This decreases (in L^∞^) the remainder terms in (6.4) and consequently allows a larger D to satisfy that inequality. From (6.7), this increases the minimum spacing between the *ψ*s and thus increases the robustness of the design. Here we have picked $\bar{H}\in [0,0.8]$ as an illustrative example.

### (a) Design

The system is designed as follows. First, for each lattice, the continuous non-dimensional geometric and material variables and the continuous dimensional parameter, *Υ*, are determined subject to the restrictions

8.1*a* $$\begin{array}{rl} & 0.1⩽\psi <1, \end{array}$$

8.1*b* $$\begin{array}{rl} & 0.25⩽\bar{\varphi }⩽4, \end{array}$$

8.1*c* $$\begin{array}{rl} & 0<{\bar{\delta }}_{\pm }⩽10, \end{array}$$

8.1*d* $$\begin{array}{rl} & -10⩽{\bar{\epsilon }}_{\pm },{\bar{\upsilon }}_{x\pm },{\bar{\upsilon }}_{xy\pm }⩽10 \end{array}$$

8.1*e* $$\begin{array}{rl} \;\mathrm{and}\; & 0<\Upsilon . \end{array}$$

(Note that (8.1*a*) does not apply to *ψ*_1_, which is not a design variable and is chosen to be 1.) These restrictions, which incorporate (3.9*a*,*b*) and (4.4*b*), ensure that the lattice system is physically feasible. However, the precise numerical values in (8.1) are arbitrary.

Note that at this stage the system is independent of length scale and material. The non-dimensional variables can be steered towards preferred regions in the design space, e.g. a preference that helices of different handedness exhibit similar bending stiffnesses, desiring $\bar{\varphi }∼1$ or requiring that the pre-strain magnitudes are similar between lattices. However, no preference has been imposed on the following examples; the restrictions in (8.1) are deemed sufficient.

In a second stage, the dimensional geometric variables, N, M and l are recovered to satisfy *ψ* (determined in the previous step) in accordance with (4.2*c*), subject to the restrictions

8.2*a* $$\begin{array}{rl} 5 & ⩽N,M⩽100 \end{array}$$

8.2*b* $$\begin{array}{rl} \;\text{and}\;50 & ⩽l⩽150, \end{array}$$

which again are arbitrary but ensure that the designs are feasible.

Length-scale dependence is introduced through l, and material dependence through the remaining physical variables d_11 ± _ and w_±_. These are chosen to satisfy the known system parameters $\bar{\varphi }$ and *Υ* for each lattice in accordance with (3.6*e*) and (4.4*a*). The material coefficients are restricted to control system feasibility; the unbounded nature of *Υ* is used to determine appropriate materials and also gives an indication of design feasibility at the interested length scale. A modified Newton's method is used to optimize over the continuous variables (Stage 1) and a genetic algorithm is used to optimize over the discrete variables (Stage 2). It should be noted that the algorithm can also be run in reverse order, i.e. given an envelope on banding numbers or/and material length or/and width restrictions, the lattice material parameters can be tuned to achieve the required system response.

### (b) Implementation

Next we highlight some details of the implementation. As demonstrated in §5, the strain energy function of a lattice system can be approximated by a truncated Taylor polynomial. The accuracy of the approximation depends on the order of the expansion, which, in turn, determines the minimum number of lattices required, as detailed in §6. Systems exhibiting complex behaviours require higher-order polynomials to approximate them to within the predefined tolerance, and consequently require more lattices. In the presence of restrictions (8.1) and (8.2) on the geometric and material parameters, the system will often need to be expanded to an order higher than the target polynomial so that the system response is within the predefined tolerance *and* the parameter restrictions are satisfied. We describe such a system as over-expanded. Example 2 (§8d) is an exactly expanded system, Example 3 (§8e) is under-expanded. Examples 1 and 4 (§8c,f, respectively) are over-expanded.

To highlight the errors associated with the framework presented in this paper, as opposed to the approximation method, we use Lagrange interpolation to generate the target polynomials. The nature of the numerical framework we have presented allows for discrete implementation of the approximation constraints; the target polynomial may be replaced by discrete target points, assuming the behaviour at locations other than the target points is not of interest. See, [32,33] for a comprehensive discussion on interpolation and approximation techniques of smooth functions by polynomials.

When it is not the energy *per se* but rather its first or second derivative (i.e. reaction force or stiffness, respectively) which are prescribed, the highest prescribed derivative is chosen to be matched where integration constants are inherently determined from the chosen material coefficients (cf. (3.8)).

We are now ready for the examples. Each of these represents only a single point in a solution space whose richness allows the designer to explore and optimize designs in accordance with requirements. The non-uniqueness of the solution space maximizes both the range of applicability of the algorithm and the physical plausibility within the design space itself.

### (c) Example 1: monostable system with prescribed nonlinear stiffness

A monostable system is designed to exhibit a nonlinear stiffness which is the third-order Lagrange polynomial of the data points

8.3 $$(\bar{H},\Xi^{\prime \prime })\in \left\{(0,1),(0.27,5),(0.53,4),(0.80,8)\right\},$$

to a tolerance of ±0.2 kN mm^−1^. A single stable equilibria exists at $\bar{H}=0$.

The system is composed of four lattices (over expansion), where an energy expansion of $O({\bar{H}}^{8})$ and a stiffness expansion of $O({\bar{H}}^{4})$ captures the prescribed stiffness function to within the specified tolerance over the entire domain. The geometric and material parameters of this system are presented in the electronic supplementary material, table S1.

**Figure 4.**
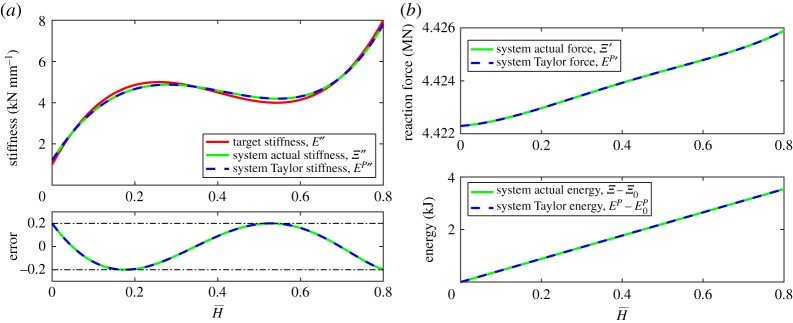
Example 1: a four-lattice monostable system with nonlinear stiffness prescribed to be the Lagrange polynomial of (8.3) with tolerance of ±0.2 kN mm^−1^. The absolute error between the actual and target stiffnesses is shown as a solid green line, and between the polynomial (Taylor) and target stiffnesses as a dashed blue line. (*a*) System stiffness and absolute error and (*b*) system reaction force and energy. (Online version in colour.)

The target stiffness, the actual stiffness, the polynomial stiffness and the absolute error in the approximation are shown in figure 4*a*. The resulting sixth-order reaction force and seventh-order monostable system energy are shown in figure 4*b*.

### (d) Example 2: bi-stable system with specified local minima

A bi-stable system is designed to exhibit stable equilibria (i) in the interior, at $\bar{H}=0.6$, and (ii) on the boundary $\bar{H}=0$, in accordance with (4.8). The second-order target reaction force is the Lagrange polynomial of the data points

8.4 $$(\bar{H},\Xi^{\prime })\in \left\{(0.25,1),(0.5,-1),(0.8,0)\right\},$$

to a tolerance of ±0.2 kN.

**Figure 5.**
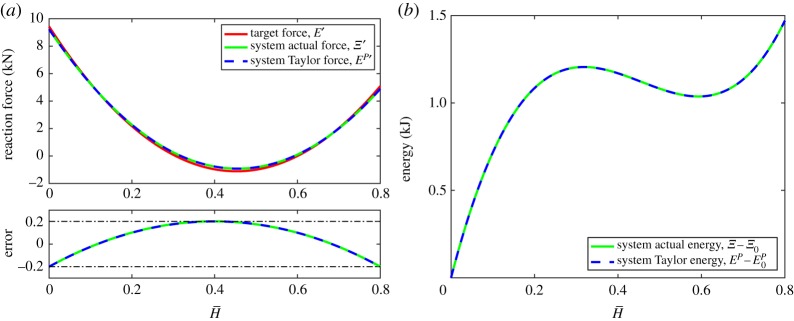
Example 2: a two-lattice bi-stable system with local minima specified to be at $\bar{H}\in \{0,0.6\}$. Target reaction force is the Lagrange polynomial of (8.4) to a tolerance of ± 0.2 kN. The absolute error between the actual and target reaction forces is shown as a solid green line, and between the polynomial (Taylor) and target reaction forces as a dashed blue line. (*a*) System reaction force and absolute error and (*b*) system energy. (Online version in colour.)

The system is exactly expanded using two lattices, the resulting second-order bi-stable target reaction force, actual reaction force, polynomial reaction force and the absolute error in the approximation are shown in figure 5*a*. The system's corresponding fourth-order bi-stable energy profiles are shown in figure 5*b*.

As discussed in §7c, the even roots of the stiffness polynomial are stable, i.e. two equilibria exist for the quadratic reaction force, one stable and one unstable, as shown in figure 5. It should be noted that due to the relatively benign nonlinearity in the system only two lattices with an expansion of $O({\bar{H}}^{4})$ exactly matching the order of the target polynomial is required to capture the $O({\bar{H}}^{3})$ target force function to within the specified tolerance over the entire domain. The geometric and material parameters of this system are presented in the electronic supplementary material, table S2.

### (e) Example 3: multi-stable system with specified local minima

Extension of the requirements to design a bi-stable system to be multi-stable is trivial within the current framework. In this example, two internal and one boundary stable equilibria are selected in accordance with equation (4.8). The target reaction force is defined as the Lagrange polynomial of the data points

**Figure 6.**
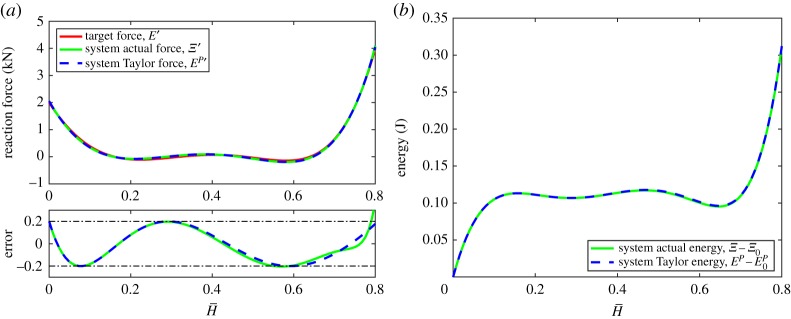
Example 3: a four-lattice multi-stable system with a nonlinear stiffness. The target reaction force is the Lagrange polynomial of (8.5) to a tolerance of ±0.05 kN, with stable equilibria specified at $\bar{H}\in \{0,0.32,0.64\}$. The absolute error between the actual and target reaction forces is shown as a solid green line, and between the polynomial (Taylor) and target reaction forces as a dashed blue line. (*a*) System reaction force and absolute error and (*b*) system energy. (Online version in colour.)

8.5 $$(\bar{H},\Xi^{\prime })\in \left\{(0,2),(0.16,0),(0.32,0),(0.48,0),(0.64,0),(0.8,4)\right\},$$

to a tolerance of ±0.05 kN, with stable equilibria specified at $\bar{H}\in \{0,0.32,0.64\}$. The system is composed of four lattices, the resulting fifth-order multi-stable target reaction force, actual reaction force, polynomial reaction force and the absolute error in the approximation are shown in figure 6*a*. The system's corresponding multi-stable energy profiles are shown in figure 6*b*.

As discussed in §7c, even roots of the stiffness polynomial are stable. The depth of the energy wells associated with the stable equilibria are particularly shallow in this example, hence the resulting transition force between the energy wells is also rather small. Consequently, a tighter tolerance has been enforced to ensure all equilibria of interest are captured. Within the current framework, it is trivial to adjust the constraints in this manner. The tighter tolerance coupled with the predefined bounds on the material parameters has required an over-expansion of $O({\bar{H}}^{8})$ to match the target reaction force $O({\bar{H}}^{6})$ to within the specified tolerance.

The geometric and material parameters of this system are presented in the electronic supplementary material, table S3. The effect of insufficient over-expansion can be observed at the extension limit, $\bar{H}=1$, and highlights the underlying assumption of the algorithm. Although the polynomial expansion can be tailored to match the target function to within a given tolerance, if the order of expansion is not sufficient to capture the actual nonlinear response over the entire domain the two responses will diverge; the polynomial response may remain within the tolerance band while the actual response does not. This behaviour is exacerbated with decreasing number of lattices (and thus expansion order) as well as over restrictive material parameter bounds, resulting in the point of divergence moving closer to the origin (expansion point), a common feature of low-order Taylor expansions.

### (f) Example 4: multi-stable system with specified snap-through loads

This example presents a multi-stable system where the transition force required to snap between stable regimes as well as their extensional position have been specified. Limit points, i.e. a local maximum in the reaction force, are defined by specifying both a root in the stiffness and the desired magnitude on the reaction force. Care must be taken to ensure this point is a local maximum.

In addition to prescribing the magnitude and location of limit points, three stable equilibria have (optionally) been specified in between them, one below the first snap-through load, $\bar{H}=0$, one (anywhere) between the two snap-through loads and one at the extension limit $\bar{H}=1$, such that the system's preferred stable configuration changes when each snap-through load is exceeded. Specifying monotonically increasing snap-through loads and their associated stable equilibria in this manner allows loading thresholds experienced by the system to be preserved upon unloading; the elastic system behaves as a reusable sensor.

The sixth-order target reaction force polynomial is defined as the Lagrange polynomial of the data points

8.6*a* $$\begin{array}{rl} \left(\bar{H},\Xi^{\prime }\right) & \in \left\{(0,0),(0.16,5),(0.48,0),(0.696,20)\right\} \end{array}$$

**Figure 7.**
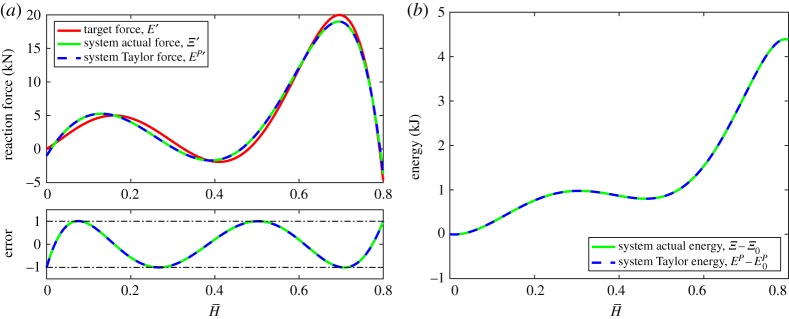
Example 4: A six-lattice multi-stable system with prescribed snap-through loads. The target reaction force is the Lagrange polynomial of the data points in (8.6) to a tolerance of ±1 kN, with an unstable equilibrium specified to exist for $\bar{H}\in \{0.16,0.696\}$. Snap-through loads have been specified as 5 ± 1 kN and 20 ± 1 kN. The absolute error between the actual and target reaction forces is shown as a solid green line, and between the polynomial (Taylor) and target reaction forces as a dashed blue line. (*a*) System reaction force and absolute error and (*b*) system energy. (Online version in colour.)

8.6*b* $$\begin{array}{rl} \;\text{and}\;(\bar{H},\Xi^{\prime \prime }) & \in \left\{(0.16,0),(0.696,0)\right\}, \end{array}$$

with the snap-through loads specified as 5 ± 1 kN and 20 ± 1 kN at $\bar{H}=0.16$ and $\bar{H}=0.696$, respectively. Accordingly, an over-expanded system of six lattices with an expansion of $O({\bar{H}}^{11})$ has been employed to capture this highly nonlinear behaviour over the entire domain to within a tolerance of ±1 kN in the reaction force, as shown in figure 7*a*. The system's corresponding multi-stable energy profiles are shown in figure 7*b*. Under the restrictions (8.1) and (8.2), a significantly higher level of expansion is needed to control the behaviour of the system to within the given tolerance over the entire domain, mitigating the effects observed in figure 6*a*. This demonstrates how increasing the nonlinearity through addition of limit points and/or raising the order of target polynomial by a single degree may require more than the addition of a single lattice so that the system is sufficiently over-expanded. Equally, a reduction in the number of lattices may be achieved through relaxation of the bounds on the material parameters. The geometric and material parameters of this system are presented in the electronic supplementary material, table S4.

## 9. Conclusion

Fully exploiting the paradigm shift from the linear-only to linear-and-nonlinear structural responses remains an open challenge. To address this challenge, and highlight the new opportunities it presents, we have developed a systematic approach to designing bespoke, elastically nonlinear, one-dimensional extensional responses. We achieve this by harnessing the behaviour of the helical lattice structure proposed by Pirrera *et al.* [18] and demonstrating how a hierarchical system comprising several lattices coupled together offers a route to obtain bespoke elasticity via a robust design process. We show how an increase in the geometric complexity of the system increases the richness in response by identifying how each unique lattice (i.e. one that possesses a unique geometric parameter *ψ* which is the scaling factor between local and global extension) added to the system extends the possible response types. This permits the formulation of a basis that spans polynomial energies to arbitrary accuracy in L^∞^. We use this framework to demonstrate how several desirable responses, such as multi-stability and snap-through buckling, can be achieved and explore the requirements for their existence.

The design space for the helical lattice system proposed here is extremely robust. The energetic description of the system permits an exploration of the interplay between pre-strain, geometry and stiffness. The non-dimensional approach allows behaviours to be considered at various geometric length scales, characteristic material stiffnesses and system curvatures. This leads to a description that is applicable for both structural (from civil to MEMS length scales) and meta-material-like requirements.

## Supplementary Material

Supplementary Data Set

## Data accessibility

The datasets supporting this article have been uploaded as electronic supplementary material.
