# Supplementary material for: Bespoke extensional elasticity through helical lattice systems
Source: Proc Math Phys Eng Sci. 2019 Dec 4;475(2232):20190547. doi: 10.1098/rspa.2019.0547 (PMC6936616; doi:10.1098/rspa.2019.0547)
Supplement: Supplementary Data Set [file rspa20190547supp1.pdf]

Bespoke Extensional Elasticity Through Helical Lattice Systems - Supplementary Material

Maximillian D. X. Dixon[1], Matthew P. O'Donnell[1], Alberto Pirrera[1] and Isaac Chenchiah[2]

[1]Bristol Composites Institute (ACCIS), Dept. of Aerospace Engineering, University of Bristol, Bristol BS8 1TR, UK
[2]School of Mathematics, University of Bristol, Bristol BS8 1TW, UK

Table S1: Geometric and material parameters for Example 1. A nominal energy value of $\Xi_0 = 264.37\,\mathrm{kJ}$ was used.

i	l	N	M	ψ	δ_+	δ_-	$\bar{\epsilon}_+$	$\bar{\epsilon}_-$	$\bar{\upsilon}_{x+}$	$\bar{\upsilon}_{x-}$	$\bar{\upsilon}_{xy+}$	$\bar{\upsilon}_{xy-}$	$\bar{\varphi}$	Υ
1	91.55	19	19	1.00	0.06	0.06	-0.22	-0.09	4.01	-1.44	-1.93	1.15	2.85	297.88
2	114.43	28	60	0.37	2.23	6.26	0.49	-1.33	4.28	-7.79	-8.48	-6.41	0.96	714.57
3	79.39	55	92	0.69	0.24	7.03	-0.44	-0.01	-2.22	-2.26	-5.34	1.58	0.25	809.58
4	97.16	56	73	0.72	1.86	0.58	-0.26	-0.44	-1.35	3.86	-2.11	6.27	0.87	764.52

Table S2: Geometric and material parameters for Example 2. A nominal energy value of $\Xi_0 = 11.961\,\mathrm{kJ}$ was used.

i	l	N	M	ψ	δ_+	δ_-	$\bar{\epsilon}_+$	$\bar{\epsilon}_-$	$\bar{\upsilon}_{x+}$	$\bar{\upsilon}_{x-}$	$\bar{\upsilon}_{xy+}$	$\bar{\upsilon}_{xy-}$	$\bar{\varphi}$	Υ
1	107.22	45	49	1.00	0.54	0.37	0.02	-0.60	-3.34	9.99	-5.81	-3.43	0.31	54.31
2	102.28	30	61	0.56	0.88	2.44	-0.94	1.56	-9.78	8.81	8.36	-3.72	4.00	21.60

Table S3: Geometric and material parameters for Example 3. A nominal energy value of $\Xi_0 = 546.59\,\mathrm{kJ}$ was used.

i	l	N	M	ψ	δ_+	δ_-	$\bar{\epsilon}_+$	$\bar{\epsilon}_-$	$\bar{\upsilon}_{x+}$	$\bar{\upsilon}_{x-}$	$\bar{\upsilon}_{xy+}$	$\bar{\upsilon}_{xy-}$	$\bar{\varphi}$	Υ
1	103.89	80	74	1.00	1.91	1.91	-0.63	-0.98	0.53	-2.92	1.54	-4.08	0.59	343.73
2	124.98	61	56	0.84	2.76	1.08	-1.57	0.08	-6.30	-5.96	6.84	-4.50	2.06	286.61
3	72.62	30	74	0.54	2.16	4.41	-0.39	-0.45	-8.30	-5.39	0.20	8.97	3.00	237.46
4	108.90	58	63	0.81	0.95	0.58	-0.09	-0.46	2.63	6.67	-0.06	-3.05	1.51	725.74

Table S4: Geometric and material parameters for Example 4. A nominal energy value of $\Xi_0 = 3.8990\,\mathrm{MJ}$ was used.

i	l	N	M	ψ	δ_+	δ_-	$\bar{\epsilon}_+$	$\bar{\epsilon}_-$	$\bar{\upsilon}_{x+}$	$\bar{\upsilon}_{x-}$	$\bar{\upsilon}_{xy+}$	$\bar{\upsilon}_{xy-}$	$\bar{\varphi}$	Υ
1	128.14	97	42	1.00	6.77	4.14	1.38	-1.56	-3.02	-0.01	1.07	-5.34	0.40	553.32
2	125.47	28	34	0.37	7.15	0.99	2.58	-0.75	-8.02	-3.77	-1.64	-5.04	3.53	8916.19
3	68.83	51	57	0.72	1.55	0.02	-1.00	0.13	-6.30	-2.58	6.81	-3.48	2.87	4088.55
4	114.58	60	34	0.86	0.14	5.65	-0.29	1.23	0.70	-1.11	-1.07	-1.21	0.25	7803.17
5	105.07	53	99	0.29	1.31	0.41	0.98	0.14	1.07	1.86	7.10	0.92	0.69	3422.46
6	116.99	98	75	0.62	0.06	0.03	0.24	-0.17	0.84	5.48	-2.17	1.97	3.97	7804.10
